# Burnout Among Orthopedic Surgeons and Surgical Trainees: A Systematic Review and Meta-Analysis of the Prevalence and Associated Factors

**DOI:** 10.7759/cureus.92830

**Published:** 2025-09-21

**Authors:** Armstrong K Nicholas, Kehinde Opashola, Omotoye Itunuola, Rupert Chima, Ridwanullah O Abdullateef, Chukwuemeka Obuekwe, Damilola Jesuyajolu, Obinna Ikegwuonu

**Affiliations:** 1 Research, Surgery Interest Group of Africa, Lagos, NGA; 2 Neurosurgery, Association of Future African Neurosurgeons, Yaounde, CMR

**Keywords:** job stress, orthopedics surgery, ortho surgery, surgeon burnout, surgical trainee

## Abstract

Orthopedic surgery is demanding and challenging. This review aimed to determine the global prevalence of burnout among orthopedic surgeons and identify the associated factors.

An extensive literature review was conducted. All full-text articles reporting data about burnout in orthopedic surgeons using the Maslach Burnout Inventory (MBI) were included. Databases such as PubMed and EMBASE were searched, and relevant data on burnout prevalence and associated factors were extracted. A random effects model was used in the prevalence meta-analysis.

Eight studies met the criteria. A total of 4006 residents and specialists were involved in all the studies. With the random effects model, the overall prevalence of burnout was estimated to be 45%. The result of the single study exclusion analysis carried out showed that no single study had a significant effect on heterogeneity. The identified factors that predisposed to burnout included medical error within the past 3 months, depression, living without a partner, absence of mandatory rest period, absence of marks of gratitude from seniors and patients, sleep deprivation, early years of training, significant other in active military duty, dissatisfaction with career choices and work-life balance, high workloads, lack of co-resident support, lack of program support and health-related issues.

Burnout is prevalent among orthopedic surgeons, and there is a disproportionate burden compared to other specialties. A problem-solving approach to addressing these factors will prove beneficial in improving the well-being of trainees. This will ultimately result in better patient care outcomes.

## Introduction and background

Orthopedic surgery is known for being arduous, demanding, and challenging. It requires a lot of sacrifices while trying to acquire the requisite clinical skills and theoretical knowledge. This puts Orthopedic surgeons at risk of a significant decline in other areas of life during training years, with the end result being burnout syndrome. Burnout is a psychological and behavioral syndrome. It involves the feeling of emotional exhaustion, depersonalization, and diminished personal accomplishment at work [1]. It is common among individuals who do highly demanding jobs and healthcare professionals [2]. The terminology burnout syndrome was initially described by the psychiatrist Freudenberger in the 1970s, which was then followed up with work by Maslach et al in the 1980s [3]. Maslach burnout inventory is the first scientifically developed measure of burnout and is a widely used tool in research studies around the world [3].

Maslach defined burnout as a tridimensional syndrome characterized by a combination of high levels of emotional exhaustion (EE), high levels of depersonalization (DP), and a low sense of personal accomplishment (PA). EE is a depletion of emotional resources through work. DP is a cynical or dehumanizing perception toward service recipients, in this case, patients. PA refers to a service provider’s perception of the efficacy or usefulness of their work. Medscape Orthopedist lifestyle recorded burnout rate of 37% among Orthopedic surgeons [4]. A study carried out in Australia recorded burnout rate in the range of 50-60% among Orthopedic surgeons, higher than what is typically seen among surgeons in general (30-40%), while a cross-sectional study on burnout syndrome among Orthopedic surgeons in Lagos state, Nigeria, recorded a burnout rate of 51.7% [5-6]. 

The major factors responsible for burnout are early years of training, working long and irregular hours, and lack of program support, and their consequences cannot be underemphasized as they greatly impact the general well-being of orthopedic surgeons and the quality of healthcare delivered to patients [7-9]. It has been linked to a myriad of physical and psychological conditions, such as insomnia, depression, substance use, alcohol abuse, suicidal ideation, and suicide. Job dissatisfaction, absenteeism, and medical errors were recognized work-related outcomes [6,10-12].

## Review

Materials and methods

Eligibility Criteria and Quality Evaluation

All full-text articles reporting data related to burnout in orthopedic surgery using the Maslach Burnout Inventory (MBI) were eligible for inclusion. Articles that did not use the MBI as the tool for measuring burnout were excluded. Meta-analyses, reviews, commentary, and poster/presentation abstracts were excluded. Articles that involved other surgical specialties were also excluded.

The risk of bias vis-à-vis the primary studies was assessed using the Newcastle Ottawa Quality Evaluation Scale (NOS) [13]. It consists of a checklist with three sections, which include study selection, group compatibility, and the outcome of interest.

Information Sources, Selection, Data Charting, and the MBI

In order to search for relevant information, we searched PubMed, Embase, and AJOL. The strategy for the search was drafted by the leading author, N.A. We used a combination of MeSH terms, keywords, and text words based on the database we searched. We focused on terms that included burnout and orthopedic surgeons.

The keywords were “burnout” AND "orthopaedic surgeons”. The search strategy can be seen in Table 2 (Appendices).

Duplicates were removed, and the titles and abstracts were evaluated by six authors. The full texts were then screened meticulously.

The relevant data were then extracted from these selected studies and used for this study. The MBI is a validated instrument used in assessing burnout and is composed of three subscales, which include emotional exhaustion, depersonalization, and low personal accomplishment. It is a 22-item questionnaire. However, there are variations of the MBI, like the validated abbreviated MBI (aMBI), which is a 9-item questionnaire that still assesses the same categories as the original MBI. Burnout is said to occur when there are high scores of EE and or DP categories with or without a low sense of personal accomplishment. Due to variations in the methods by which the individual studies identified the factors affecting burnout, we were unable to carry out a quantitative analysis. However, after going through all the papers, we did a narrative synthesis of the factors affecting burnout as identified by each study and also discussed them.

Statistical Analysis

A random effect was used for the statistical analysis. This was done using the Meta XL add-on for Microsoft Excel with the use of the double arcsine transformation. The random effect was used to account for heterogeneity. Prevalence was calculated at a confidence interval of 95%.

We did a subgroup analysis of studies that had a high response rate (50% or more) compared to studies with low response rates. And in addition, a subgroup analysis of high-quality studies based on the NOS (7 and above) was conducted [13].

Results

PRISMA Flowchart

**Figure 1:**
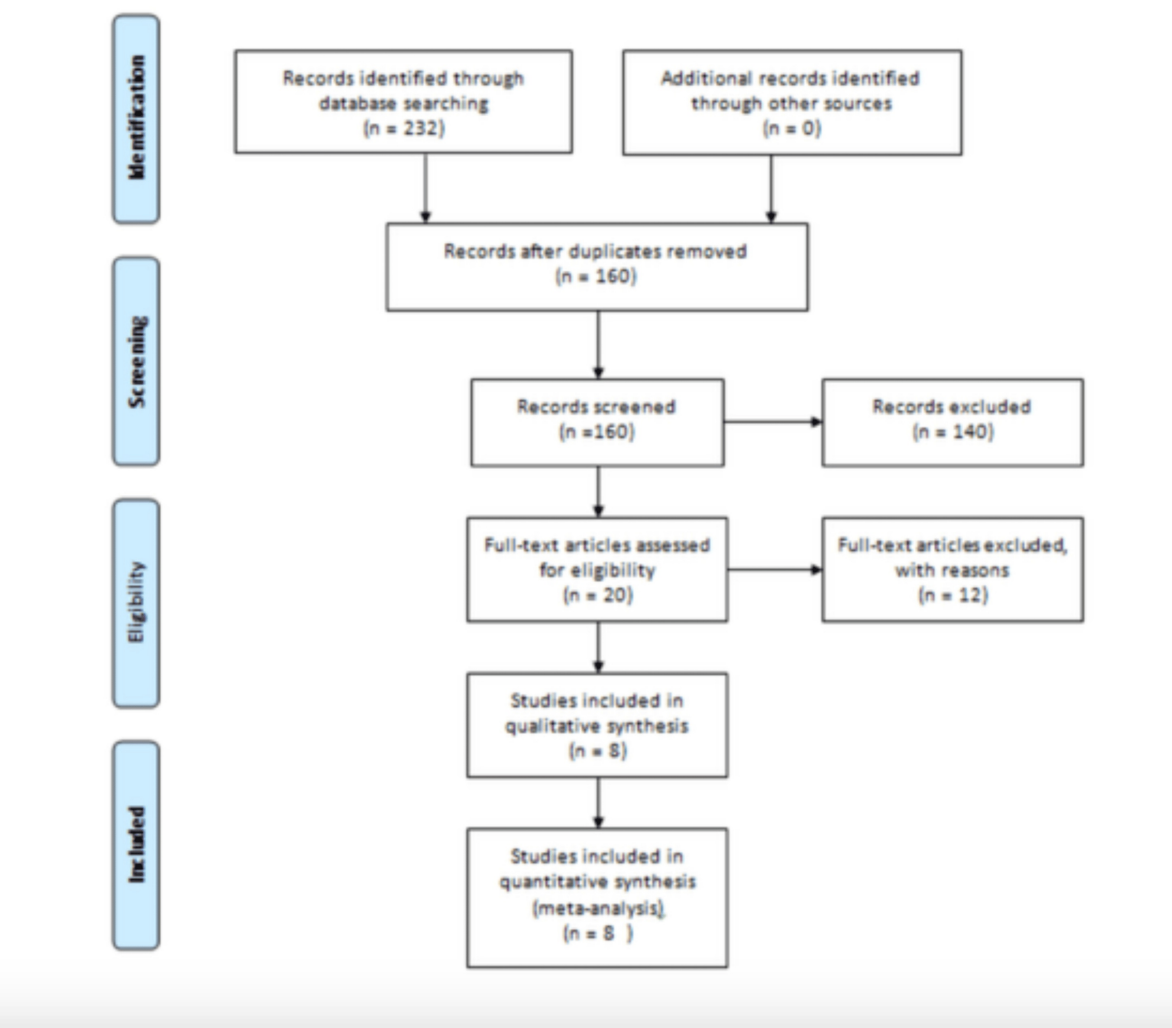
Prisma flow diagram of the study

The Prisma flowchart is shown in Figure 1. A total of 232 studies were identified, and a total of 160 studies remained after removal of duplicates. One hundred forty studies were excluded after titles and abstracts were screened. Twenty papers were initially selected for qualitative synthesis, but were further trimmed down to 8. This was following a team discussion where it was discovered that 10 studies used other non-validated instruments to evaluate burnout aside from the MBI, while 2 did not provide adequate information regarding the total number of residents approached with questionnaires and response rates.

NOS Evaluation

The eight selected studies for this study were submitted to the Newcastle Ottawa Quality Evaluation Scale (NOS) to determine the risk of bias, as seen in Table 1 [13]. Four studies scored 8, two studies scored 7, while one study scored 5 [5-9,14-15].

**Table 1:** Summary of studies considered in the analysis and burnout rates

Study	Country	Population	Response Rate	Burnout Rate (n)	Respondents	Male	NOS
Faivre et al. (2018) [14]	France	Residents	22%	72% (77)	107	65%	8
Simons et al. (2016) [7]	United States	Residents and Specialists	67%	7.7% (3)	39	NA	8
Arora et al. (2014) [5]	Australia	Residents	22%	53% (27)	51	86%	8
Van Vendeloo et al. (2014) [8]	Netherlands	Residents	94%	27.6% (29)	105	79%	8
Faivre et al. (2019) [15]	France	Specialists	23%	49.6% (219)	441	93.7%	7
Lichstein et al. (2020) [9]	United States	Residents	58%	52% (342)	661	83.4%	7
Ho and Kwek (2022) [16]	Singapore	Residents	100%	45.5% (20)	44	90.9%	7
Coker et al. (2012) [6]	Nigeria	Residents and Specialists	100%	51.7% (15)	29	96.6%	5

The Overall Prevalence of Burnout in Orthopedic Surgery

**Figure 2:**
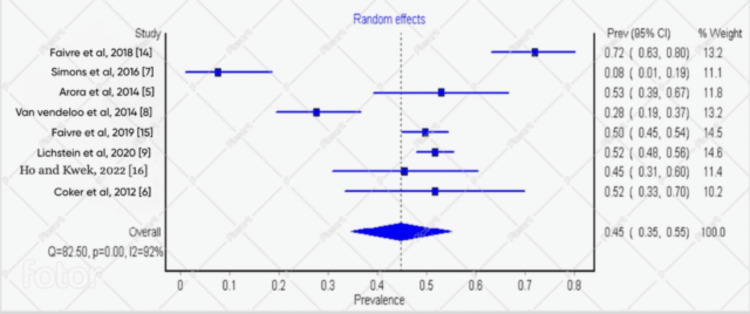
Forest plot showing the overall prevalence of burnout in orthopedic surgery

The eight included studies involved about 4006 residents and specialists. Of the 4006 surgeons, 732 met the criteria for burnout (Table 1). With the random effects model, the overall prevalence of burnout in orthopedic surgery was estimated to be 45% (Figure 2).

Factors Associated with Burnout

The factors associated with orthopedic burnout from the studies include Medical error within the past 3 months, depression, living without a partner, absence of mandatory rest period, absence of marks of gratitude from seniors and patients, sleep deprivation, early years of training, significant other in active military duty, dissatisfaction with career choices and work-life balance, high workloads, lack of co-resident support, lack of program support, inability to attend health maintenance support [5,7,8,14,15].

Protective factors identified include having a mentor, having time for hobbies, social activities, spouse or child-centric group activities, improvements in the climate of learning, peer collaboration among trainees, and career advancement [5,8,9,14,15].

Sensitivity and Subgroup Analysis

**Figure 3:**
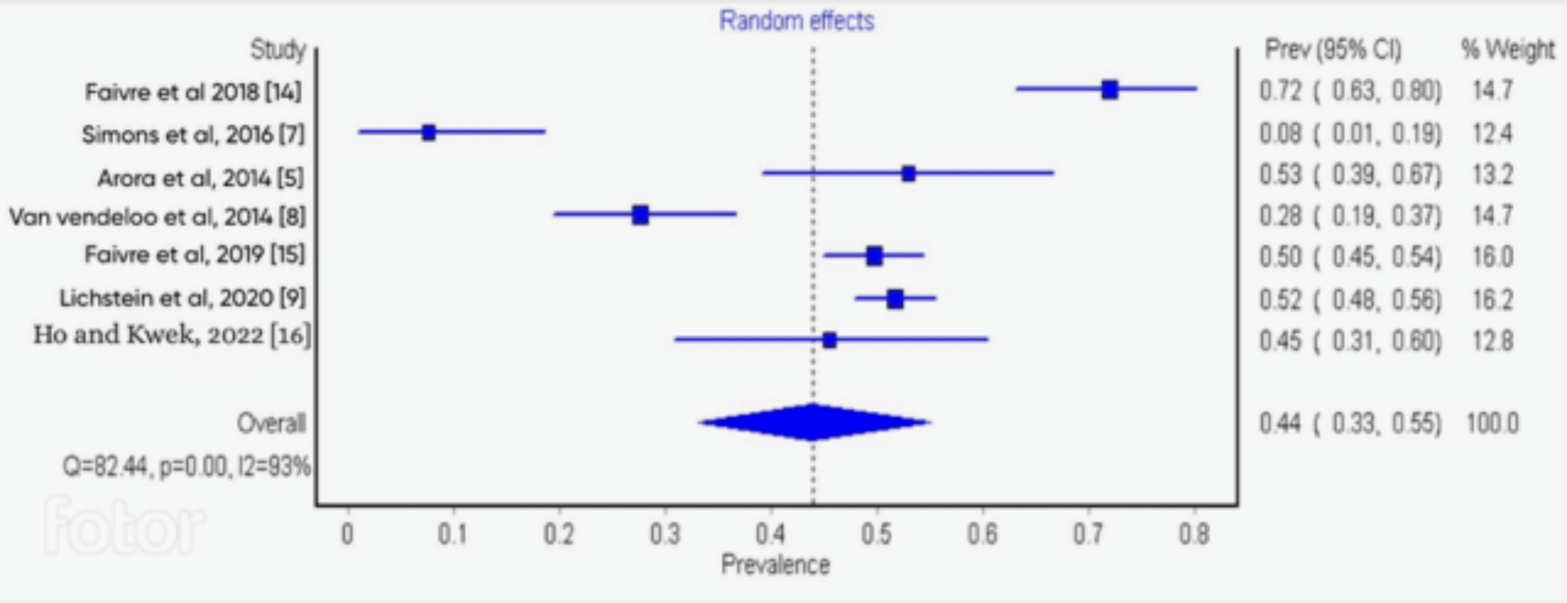
Forest plot study showing the single study exclusion sensitivity analysis, showing high-quality studies

**Figure 4:**
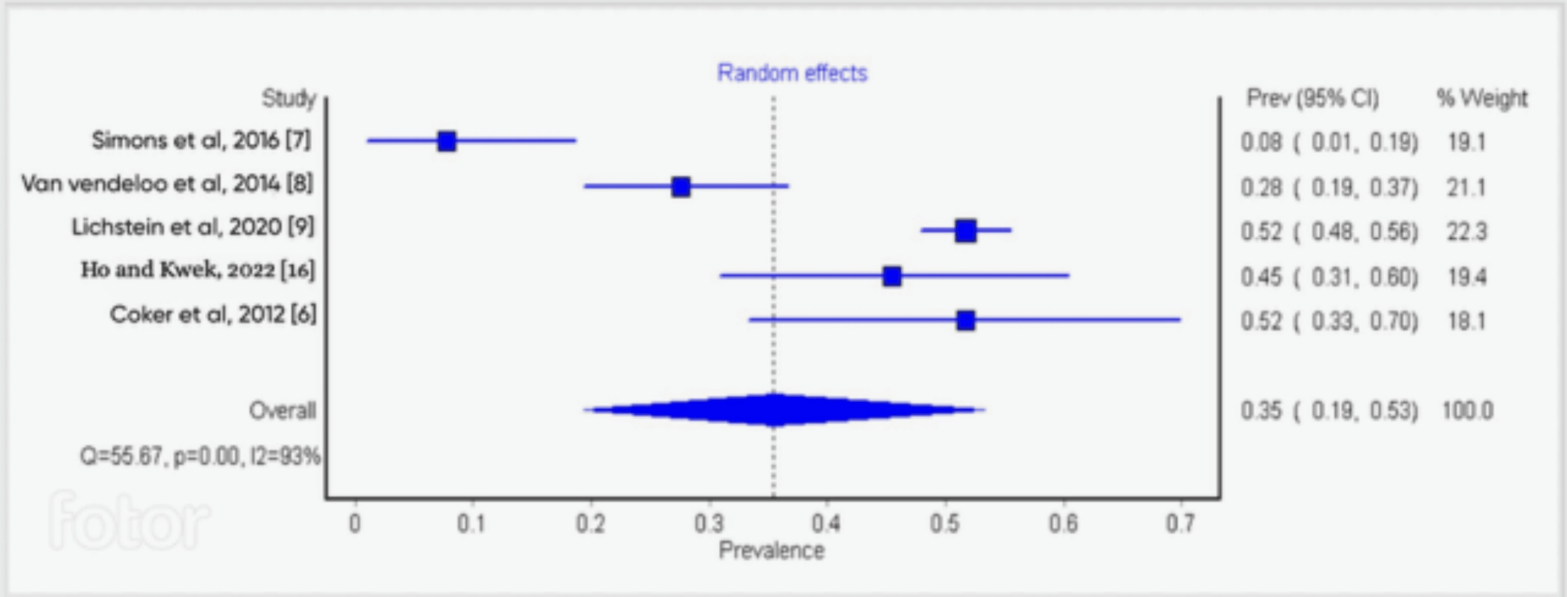
Forest plot study showing subgroup analysis of studies based on their response rates (high)

**Figure 5:**
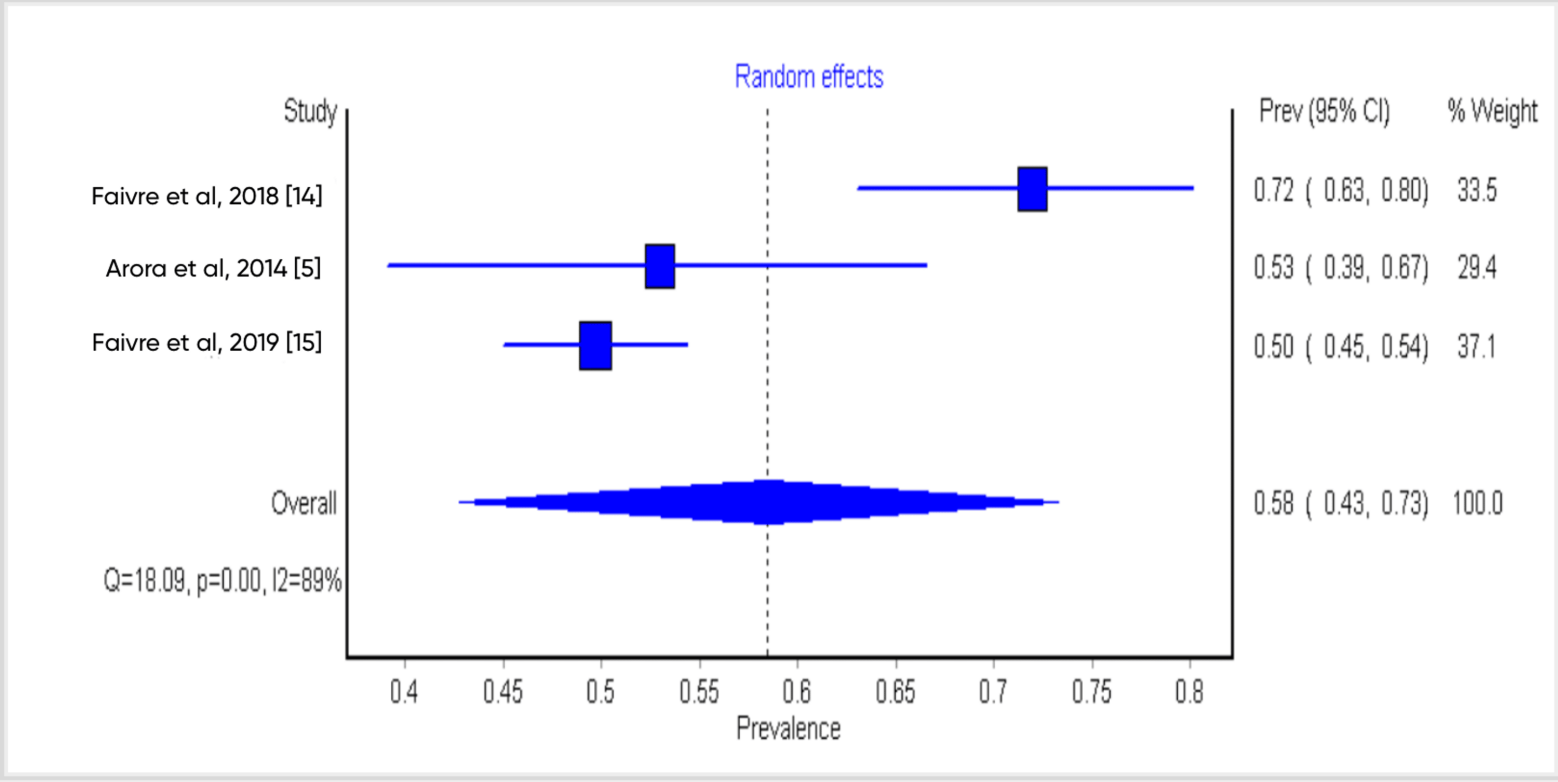
Forest plot study showing subgroup analysis based on their response rates (low)

The result of the single study exclusion analysis carried out showed that no single study had a significant effect on heterogeneity. The cumulative-study exclusion sensitivity analysis involving studies that were high quality per NOS scale (7 and above) gave an overall burnout rate of 44%. The subgroup analysis of studies based on response rates (high vs low) showed significant differences (35% vs 58%) (Figures 3-5).

Discussion

This study involved eight previous studies that met our criteria using the MBI scale. The overall prevalence of burnout among orthopedic surgeons, which included the residents and the specialists, was 45%. This is similar to the study done by Coker et al. in Nigeria in 2012 among residents and specialists in orthopedics, which had a prevalence of 51.7% [6]. However, a study conducted by Simons et al. in 2016 among orthopedic surgeons in the United States revealed a 7.7% burnout rate [7]. This could be because 39 surgeons participated in the study, which wasn’t representative enough. This value is far away from a study conducted by Lichstein et al., done in the United States in 2020, which involved 661 residents showing that 52% of orthopedic surgeons who participated in the study were burnt out [9]. The MBI scale was used; it is scored using a 7-level frequency rating from “never” to “daily”. The MBI has three component scales: emotional exhaustion (9 items), depersonalization (5 items), and personal achievement (8 items).

Factors associated with burnout

There are various risk factors that have been linked to burnout among orthopedic surgeons. The factors which were reported in the articles reviewed in this study include, medical error within the past 3 months, depression, living without a partner, absence of mandatory rest period, absence of marks of gratitude from seniors and patients, sleep deprivation, early years of training, significant other in active military duty, dissatisfaction with career choices and work-life balance, high workloads, lack of resident support, lack of program support, inability to attend health maintenance support [5,7-9,14,15].

Young surgeons and those in early years of training with lesser experience had higher risks of burnout as opposed to those who had more experience [7,9,15]. Sleep deprivation was reported as an associated risk factor; however, Lichstein et al found no association between sleep and burnout [9,16]. Arora et al. reported dissatisfaction with career and work-life balance to be linked to a higher incidence of burnout among orthopedic trainees [5]. Depression is a recognized factor in three of the studies [9,14,15]. The study carried out by Faivre et al. reported that depression is implicated as both a cause and consequence of burnout [15]. Living without a partner was another factor found to be related to burnout; however, some studies found no correlation between relationship or marital status and burnout [7,14,16]. Occupational-related factors found to increase the rate of burnout among orthopedic surgeons include working excessive hours, high volume of workload, medical errors, litigation, lack of co-resident and program support, and financial pressure [9,14,15].

Certain protective factors associated with burnout among orthopedic surgeons were also identified in this review. The male gender was recognized in two studies as a protective factor, although Simons et al. and Ho and Kwek found no significant correlation between gender and burnout [7,14-16]. It has been recommended that protective actions should be adopted among orthopedic surgeons. It is important that there should be standardized working hours to reduce the stress among residents. Creating opportunities for residents to collaborate among peers, improving the climate of learning, and having mentors will further reduce the level of burnout among orthopedic surgeons. Also, encouraging residents to pick up a hobby and practice during leisure time. It is important for there to be a department of occupational psychologists and therapists at the workplace to help alleviate the level of stress that surgeons experience. Several studies discussing the implementation of coping strategies and improving the overall well-being of surgeons have been shown to positively affect the personal and professional lives of the participants.

Study limitations

We acknowledge the following limitations. Like most systematic reviews, we relied on a relatively limited number of databases for the identification of potentially eligible studies. While this may preclude the screening of all relevant studies, it is worth mentioning that the studies included in this review were retrieved from major databases with extensive repositories. One other limitation of our review is that many of the studies included a cross-sectional and correlational study design, which precludes inference of causation. In addition, regression methods were not used to explore between-study heterogeneity. Finally, this review did not provide detailed information on the study design characteristics and demographics of participants.

## Conclusions

Burnout is highly prevalent in orthopedic surgeons and surgical trainees suffer a disproportionate burden of burnout compared to specialists. The high burnout rate among orthopedic surgery trainees is of great concern. This is because the effects of burnout may adversely affect healthcare delivery and outcomes, as these trainees are often the first contact for patients seeking healthcare in tertiary centers. It is worth noting that most of the associated risk factors for burnout are potentially modifiable. A problem-solving-based approach to addressing these factors will prove beneficial in improving the well-being of trainees and result in better patient care outcomes in the long run.

To adequately address burnout in residency programs, there is a need to assess the burden of burnout and to determine the specific risk factors driving burnout in vulnerable populations. One way of doing this is to employ the use of a validated tool such as the Maslach Burnout Inventory to measure both the extent and pattern of burnout and identify the likely causes. Emphasis should be placed on identifying and alleviating the modifiable risk factors of burnout while improving the overall well-being of trainees. Efforts should also be geared towards educating residents on burnout early in the early years of training, as well as encouraging peer support and peer advocacy. It is imperative that programs focus on preventive strategies. The best strategies are aimed at preventing burnout at personal (by raising burnout awareness and identifying trainees at risk), Institutional (by cultivating a culture of advocacy, personal responsibility, and access to physician-centered mental health care), and national levels (including favorable government policies on work hour restrictions).

## Appendices

**Table 2:** Search strategy

Keywords/MeSH Terms	Results	Database
(Burnout) AND (Orthopaedic surgeons)	161	PubMed
(Burnout) AND (orthopaedic surgeons)	48 (first two pages)	AJOL
(Burnout* AND Orthopaedic surgeons).mp.	23	Embase
